# Endodontic treatment of two-canalled maxillary central and lateral incisors: a case report

**Published:** 2009-04-17

**Authors:** Noushin Shokouhinejad, Mohammad Saeed Sheykhrezaee, Hadi Assadian

**Affiliations:** 1 Department of Endodontics, Faculty of Dentistry/Dental Research Center, Tehran University of Medical Sciences, and Iranian Center for Endodontic Research, Tehran, Iran; 2 Department of Endodontics, Faculty of Dentistry/Dental Research Center, Tehran University of Medical Sciences, Tehran, Iran; 3 Department of Endodontics, Faculty of Dentistry, Tehran University of Medical Sciences, Tehran, Iran

**Keywords:** Endodontic treatment, Extra canals, Incisor, Maxilla

## Abstract

Familiarity with the intricacies and variations of root canal morphology is essential for successful endodontic treatment. Maxillary central and lateral incisors are known to be single-rooted with one canal, however, this case report describes endodontic treatment of maxillary central and lateral incisors with two buccopalatal root canals.

## Introduction

The main objective of root canal treatment is thorough cleaning and shaping of the root canal system followed by complete obturation. One of the most important prerequisites for a successful endodontic treatment is the thorough knowledge of root canal anatomy. Maxillary incisors have widely been depicted with a single root canal within a single root (1-4); however some authors have reported maxillary central and lateral incisors with two canals and even two roots (5-16). 

This case report describes a maxillary central incisor with two roots and a lateral incisor with two canals located buccopalatally.

## Case Report

A 12-year-old girl with a non-contributory medical history was referred to Department of Endodontics, Dental School, Tehran University of Medical Sciences. The patient was asymptomatic with poor oral hygiene and no periodontal pocket. Interdental papillae of maxillary right central and lateral incisors (teeth #7 and 8) were inflamed. Both teeth had hypoplastic crowns (Figure 1). Tooth #8 had a temporary filling with fractured incisal edge. There were no swelling or sinus tract and no history of trauma. Both teeth did not respond to thermal and electrical vitality tests, palpation and percussion. Teeth #9 and 10 (controls) react normally to thermal and electric tests. Radiographic evaluation of the teeth showed rarefactions peri-apically (Figure 2).

Furthermore, there was evidence of additional canals and/or roots radiographically. Overall, final diagnosis was pulp necrosis and chronic apical periodontitis.

After scaling and root planning, tooth #8 was anesthetized (Lidocaine 2% with epinephrine 1:80000; Daroupakhsh, Tehran, Iran) and isolated with rubber dam; dental floss and wooden wedges were used to stabilize rubber dam. After access cavity preparation, two separate labial and palatal orifices were found. The working length was determined using Root ZX electronic apex locator (J Morita Corp., Kyoto, Japan) and then verified radiographically (Figure 3). The root canals were prepared using hand instruments (K-file, Mani, Japan) and Gates Glidden burs (Dentsply Maillefer, Ballaigues, Switzerland) using passive step-back technique. NaOCl 2.5% was used as an irrigant. The canals were dressed with Ca(OH)_2_ and sealed coronally with Cavit(ESPE, Seefeld, Germany). Eight days later, canals were obturated with AH26 silver free sealer (Dentsply, DeTrey, Konstanz, Germany) and laterally condensed with gutta-percha (Aryadent, Tehran, Iran). Obturation quality was confirmed radiographically (Figure 4).

**Figures:**
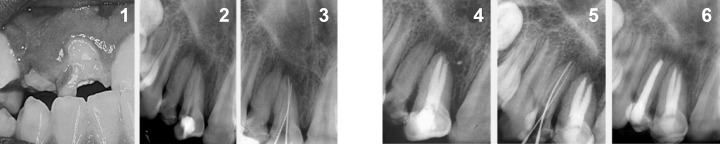
1) Photographic image of teeth #7 and #8, 2) radiographic image of teeth #7 and #8, 3) working length measurement of tooth #8, 4) postoperative radiograph of tooth #8, 5) working length measurement of tooth #7, 6) postoperative radiograph of teeth #7 and #8

At the following appointment, RCT of tooth #8 was performed similar to tooth #7. During canal preparation, the septum between two canals was removed (Figure 5-6).

## Discussion

Maxillary incisors are commonly single-rooted with one canal (1-4). Many of two-rooted cases have anomalies known as fusion or gemination, in which canals usually located mesiodistally (5,7-10,12). In this case, two root canals were situated buccopalatally. The central incisor had two separate roots and canals but the lateral incisor had a single root containing two buccopalatal root canals that merged into one wide root canal**.** Except a few reports (13,15), in two-canalled maxillary incisors, the root canals were located mesiodistally (5-12,14,16). 

Noteworthy is that the crowns of teeth #7 and 8 were hypoplastic compared to the contralateral side. Moreover, the crown of tooth #7 was somewhat dilacerated in radiography. Infection of or trauma to primary teeth can result in hypoplasia in succedaneous teeth. The crowns of teeth with gemination and/or fusion are known to be wider; however our case did not show a mesiodistally wider dimension than the contralateral side. 

## Conclusion

In conclusion, the clinician should be always attentive to detect anatomic anomalies. Importance of careful preoperative evaluation cannot be over emphasized.
